# Anthropogenic Habitat Loss and Fragmentation May Alter Coevolutionary Progress as Examined in a Brood Parasitism Model

**DOI:** 10.1002/ece3.71721

**Published:** 2025-07-16

**Authors:** Wei Wang, Timothy Van Deelen, Fuwen Wei, Sheng Li, Luping Wang

**Affiliations:** ^1^ Department of Forest and Wildlife Ecology University of Wisconsin‐Madison Madison Wisconsin USA; ^2^ State Key Laboratory of Gene Function and Modulation Research, School of Life Sciences Peking University Beijing China; ^3^ Department of Civil and Environmental Engineering University of Wisconsin‐Madison Madison Wisconsin USA; ^4^ College of Forestry Jiangxi Agricultural University Nanchang China; ^5^ Key Laboratory of Animal Ecology and Conservation Biology, Institute of Zoology Chinese Academy of Sciences Beijing China; ^6^ University of Chinese Academy of Sciences Beijing China; ^7^ Center for Excellence in Animal Evolution and Genetics Chinese Academy of Sciences Kunming China

**Keywords:** behavior adaptation, brood parasitism, coevolution, habitat loss and fragmentation, stochastic simulation

## Abstract

Habitat loss and fragmentation (HLF) resulting from anthropogenic disturbances is one of the greatest threats to numerous threatened taxa facing extinction risks. HLF may devastate biodiversity through various pathways such as restricting animal movement and gene flow, reducing opportunities for species to expand or shift their ranges and thus optimizing habitat use, and directly causing population decline and range contraction. Despite these well‐documented impacts, the effects of HLF on the coevolutionary processes between coexisting species are rarely examined. In this study, we constructed a cuckoo–host brood parasitism model to explore how HLF of varied degrees may affect the cuckoo–host population dynamics through stochastic and reinforcement simulations. The results, validated with empirical data, revealed that severe HLF significantly increases the cuckoo's extinction risk compared to moderate HLF. Furthermore, severe HLF narrows the range of host rejection rates that allow cuckoo populations to persist under natural conditions. These findings suggest that severe HLF, typically driven by human activities and anthropogenic land use change, may not only directly increase the extinction risk of specific species but also disrupt the coevolutionary interactions, posing more severe ecological consequences than previously anticipated.

## Graphical abstract

Our study explores how habitat fragmentation affects the coevolutionary dynamics between brood parasites (cuckoos) and their hosts using a stochastic, reinforcement‐based model. By incorporating both inherited traits and learned behaviors, our findings reveal how anthropogenic changes disrupt coevolutionary stability, threatening species interactions. This work provides a novel modeling framework for studying evolutionary and ecological dynamics under environmental pressures.

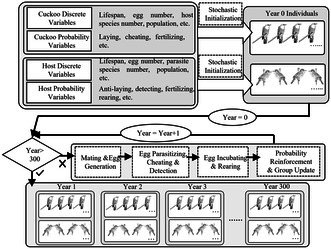

## 1 Introduction

Habitat loss and fragmentation (HLF) is a critical threat to wildlife populations, ecological communities, and global ecosystems (Andrén and Andren 1994; Hanski 2011; Haddad et al. 2015; Fletcher et al. 2018). Although HLF could be caused by natural events such as flood, wildfire, earthquake, and landslide at varied spatial scales, most HLF in the Anthropocene is associated with human activities and expanding human land use across all continents (Didham et al. 2012; Taubert et al. 2018). Human‐mediated HLF has been proven to pose profound threats to numerous species across various taxa, leading to increased extinction risks (Schipper et al. 2008; Fletcher et al. 2018). Such impacts may be derived through both direct and indirect pathways such as directly causing population decline and range contraction, restricting animal movement and gene flow, and reducing opportunities for species to expand or shift their ranges and thus optimize habitat use, etc. (Hanski 2011; Smith et al. 2011; De Camargo et al. 2018; Fahrig et al. 2019). However, the effects of HLF on the coevolutionary processes between coexisting species and their mutualistic or antagonistic interactions, a possible but previously neglected pathway through which HLF may negatively affect biodiversity (Fontúrbel and Murúa 2014), are rarely examined. In landscapes undergoing substantial habitat changes, two or more sympatric species engaged in coevolution may exhibit different responses to HLF, potentially further disrupting the interspecific disruptions (Hoover and Robinson 2007; Fontúrbel and Murúa 2014; Bitters et al. 2022; Perrin et al. 2023). Therefore, determining the patterns and mechanisms of how human‐mediated HLF affects such coevolutionary interspecific relationships and dynamics is critically needed to improve our understanding of the ecological consequences of HLF and to guide our conservation efforts accordingly.

Due to the slow march of evolutionary processes, observing coevolutionary progress over generations and in its natural settings is difficult. However, the severe and rapid HLF in the era of the Anthropocene, typically driven by human activities and anthropogenic land use change (Didham et al. 2012; Taubert et al. 2018), may pose substantial threats to the species pairs or groups engaged in coevolution, indicating an urgent need for alternative approaches beyond field studies. Here, we constructed a cuckoo–host brood parasitism simulation model, validated with empirical data, as an alternative approach to quantitatively examine the impacts of HLF on such fragile coevolutionary progress. Obligatory avian brood parasitism, found in approximately 1% of bird species, is a well‐studied and classic example of coevolution (Rothstein 1990; Winfree 1999; Davies 2010). This reproductive strategy occurs when a bird species foists the duty and cost of building nests, incubating eggs, and rearing offspring onto a host species, and the brood parasite is otherwise unable to reproduce (Rothstein 1990; Winfree 1999; Feeney et al. 2014). Previous studies have suggested that a coevolutionary arms race exists between brood parasitic birds, such as old‐world cuckoos and their hosts. In this race, each competing species (parasite and host) drives the other to become more adaptable across all reproductive stages, from nest building to fledging (Brooke and Davies 1988; Langmore et al. 2003; Welbergen and Davies 2009; De Mársico et al. 2012). During this process, each party competes with the other to increase its fitness, but neither party is forced to extinction. A critical factor in maintaining this fragile equilibrium is the host's adaptable range of rejection rates (RR) to exotic egg they may recognize based on its morphological traits, such as color and shape, which prevents one species from gaining a decisive advantage over the other (Yang et al. 2010; Spottiswoode and Stevens 2011).

In this study, we designed a simulation‐based framework to examine how and to what extent HLF alters the coevolutionary progress between cuckoos and their hosts. In this framework, we considered the reasonable RR values that ensure no extinction for both host and parasite may be determined by the proportion of suitable habitat, as random sample hypothesis proposes (Connor and McCoy 1979). If the old RR in any given habitat failed to keep the coevolutionary arm race at equilibrium—due to moderate or severe HLF altering the proportion of suitable habitat—then either a new RR value generated by natural selection would be established or one or both species would go extinct. Herein, we predicted that an adaptable range of RRs relates to the degree of HLF (moderate and slow vs severe and rapid). We also hypothesized that lower reproductive profit in the smaller patches generated by accelerated HLF may decrease the potential of a parasitic avian species to adjust its parasitism behavior, thus driving it to both find new host species and forego adapting to environmental change.

To explore these hypotheses, we developed an individual‐based simulation model that integrates stochastic inheritance and reinforcement learning to reflect both genetic and experiential factors in the coevolutionary dynamics between common cuckoos ( *Cuculus canorus* ) and their hosts. The model was designed to assess how varying levels of habitat loss and fragmentation alter population trajectories, constrain adaptive behavioral strategies, and destabilize recognition–rejection (RR) patterns over time. Grounded in empirical data, this approach allows us to examine how shifts in habitat configuration influence the persistence and flexibility of parasitic behaviors, such as the adoption of “back‐up” strategies. By simulating long‐term interactions under both moderate and severe HLF scenarios, this study aimed to offer a novel perspective on how environmental change can disrupt behavioral evolution and species coexistence.

## 2 Materials and Methods

**FIGURE 1:**
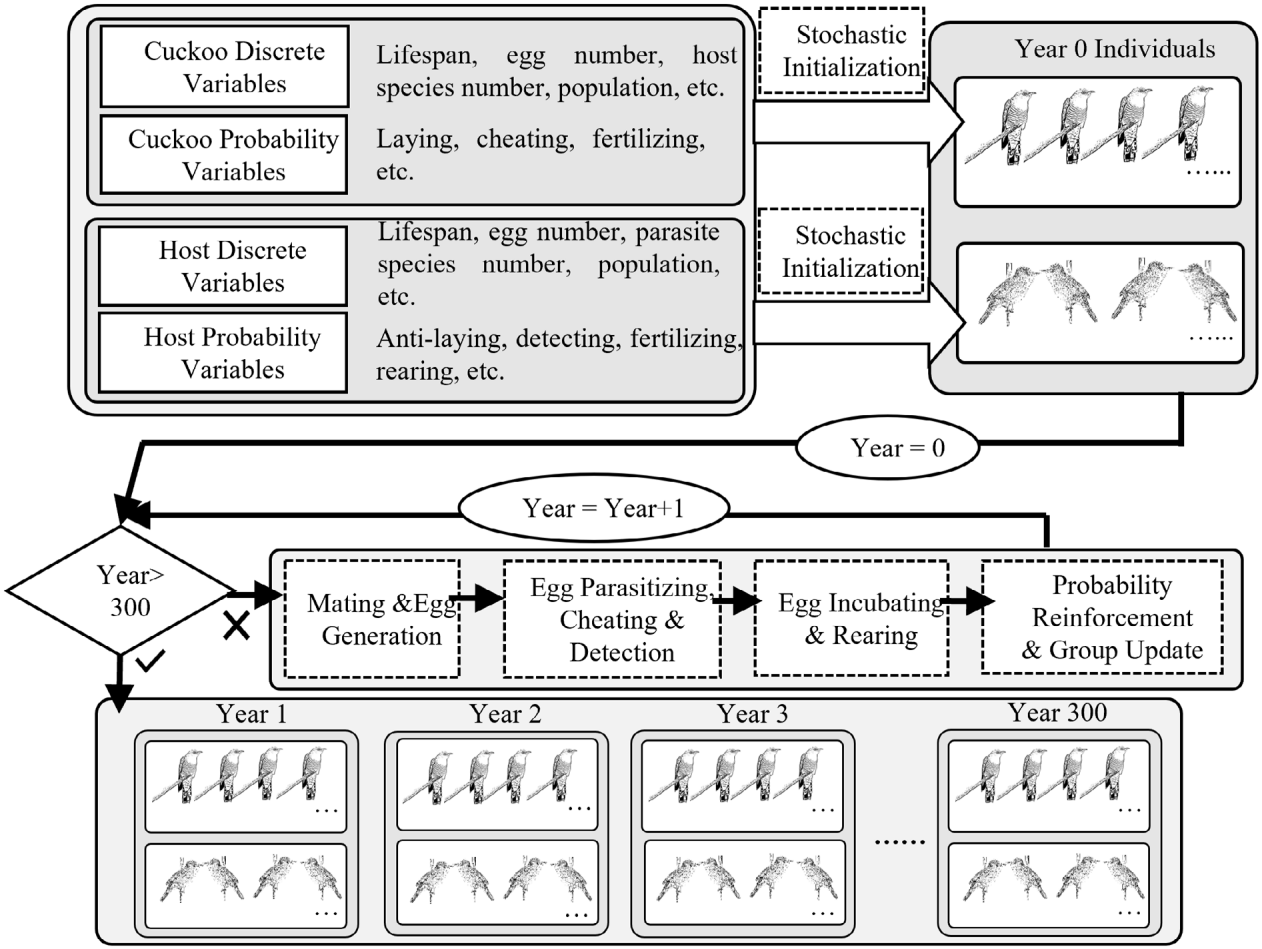
Conceptual illustration of the Stochastic Reinforcement Model. Discrete and probability parameters were used to stochastically initialize both cuckoo and host individuals. Then, models for cuckoo–host gaming are iterated for 300 years to simulate mating, egg laying, parasitizing, cheating, detection, incubating, and rearing with sequential reinforcement.

The model (Figure 1) comprised a stochastic component to simulate inherited traits and a reinforcement component to represent the learning process through which individuals gained experience over successive periods.

### 2.1 Model Parameters and Assumptions

To simulate the brood parasitism processes between cuckoos and hosts, we first initialized the parameters used in the model. For cuckoo groups, the model parameters included lifespan, egg number, host species number, initial population, and probabilities associated with laying eggs, deceiving hosts (egg color and shape), and successful fertilization. For host groups, the model parameters included lifespan, egg number, parasite species number, initial and maximum population sizes, and probabilities related to antiparasitism behaviors, parasite detection (egg color and shape), fertilization, and chick rearing. Full definitions of all parameters are provided in Table S1. To incorporate natural variability, we applied four types of stochastic processes: categorical variables (e.g., host species) were sampled from uniform distributions; probabilistic parameters (e.g., parasitism success and hatching rates) followed truncated normal distributions; long‐tailed discrete variables (e.g., lifespan) were modeled using truncated Weibull distributions; and other discrete variables (e.g., egg number) followed truncated Poisson distributions. Sampling from these distributions was carried out using the acceptance–rejection method. Detailed procedures for stochastic initialization are provided in Algorithms S1–S3 in Appendix S1. Based on these methods, we constructed several virtual cuckoo groups and host populations. For all groups, parameters for every individual of each virtual group were assigned based on population‐level values, along with small variations to reflect natural randomness. Once the initial populations were established, the simulation proceeded iteratively through the following processes over a given number of years (Algorithm S4 in Appendix S1).

### 2.2 Mating and Egg Generation Processes

**FIGURE 2:**
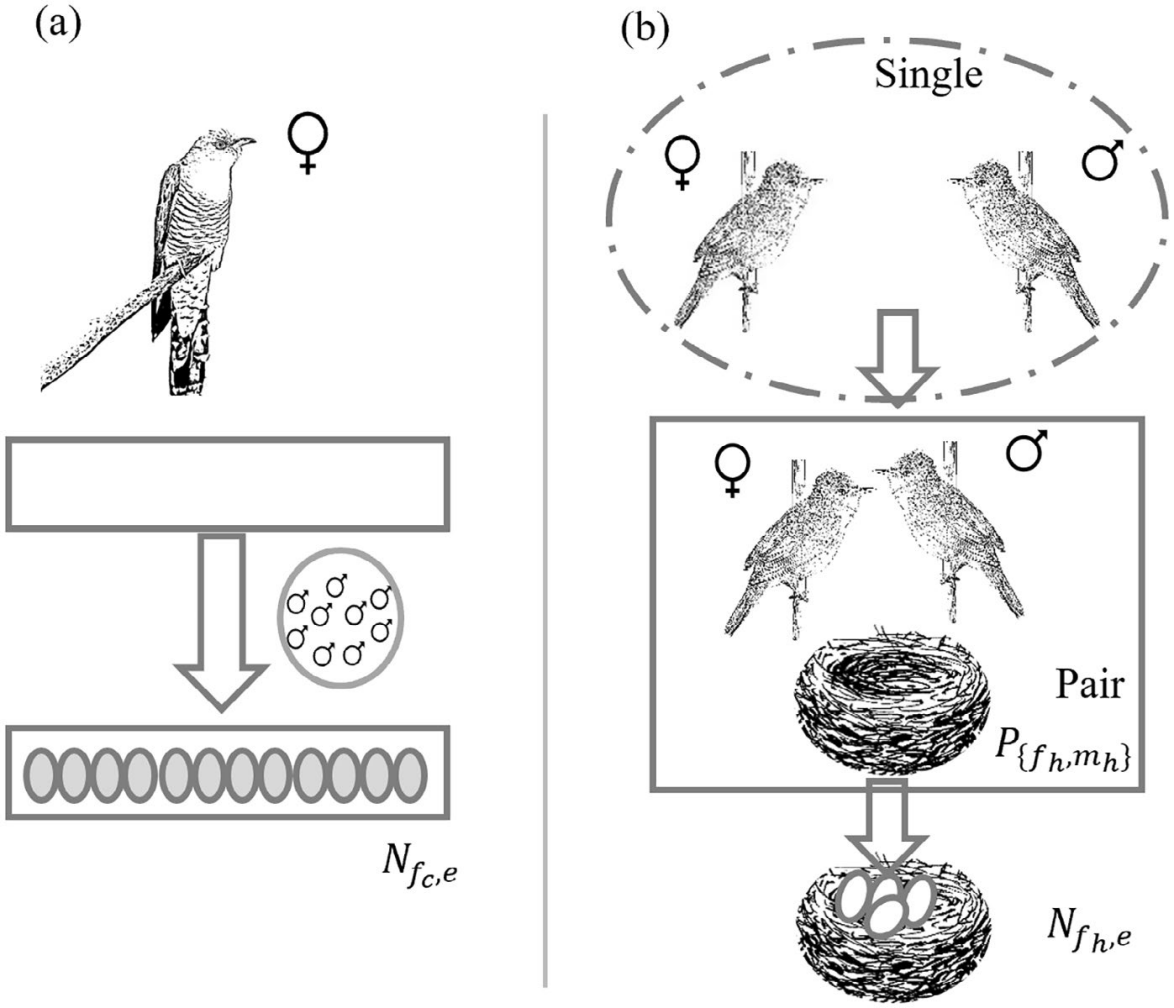
Mating and egg generation phase simulation. (a) Cuckoo behaviors: Female cuckoos mate with multiple males to generate an egg list. (b) Host behaviors: Host birds pair, construct a nest, and lay eggs.

For the mating and egg generation processes, we assumed that cuckoos followed a polygamous reproductive strategy (Meiklejohn 1917), where each female cuckoo ($f_{c}$) mated with multiple males, producing eggs up to her egg capacity ($N_{f_{c},e}$, the left part of Figure 2). In contrast, host species were assumed to use a monogamous reproductive strategy (Davies et al. 2003), with host parents randomly paired to form reproductive units ($P_{h}$, or $P_{\left\{f_{h},m_{h}\right\}}$, where h means host, $f_{h}$ and $m_{h}$ are the female and male host, respectively). Thereafter, each unit pair built a nest and laid $N_{f_{h},e}$ eggs in it (the right part of Figure 2). Egg generation followed stochastic inheritance rules to assign parameters values for offsprings: probabilistic variables were modeled using truncated normal distributions, while long‐tailed discrete variables (e.g., lifespan) followed truncated Weibull distributions. For maternally inherited traits, such as parasite egg color and shape, the expected value in the offspring was determined solely by the female parent. For other traits, the expected value was computed as the average value between both parents. Detailed procedures for mating and egg generation are described in Algorithms S5 and S6 in Appendix S1.

### 2.3 Egg Parasitizing Processes

**FIGURE 3:**
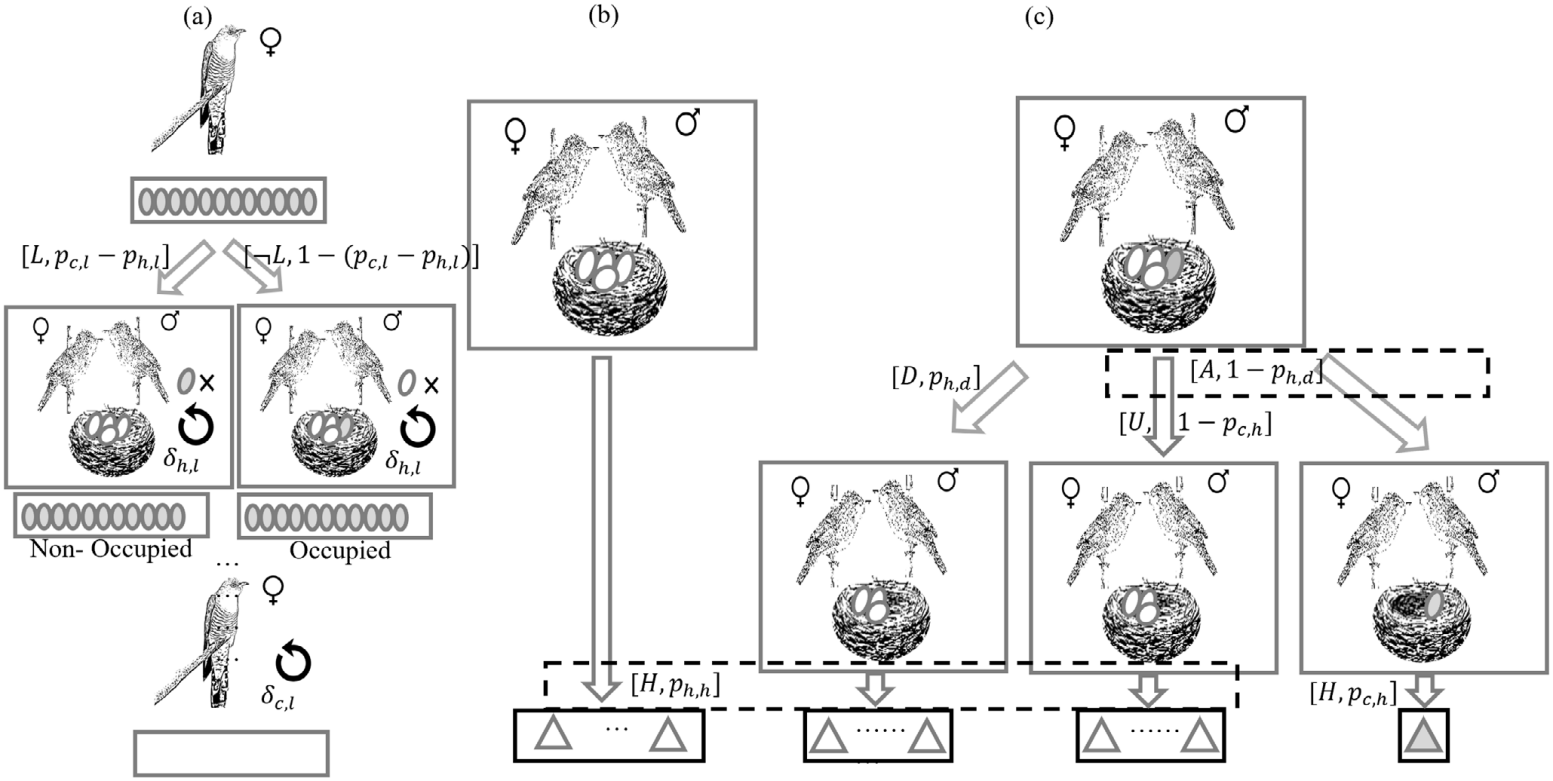
Parasitizing, cheating, detection, and incubating simulation. (a) presents cuckoo behaviors. Female cuckoos (*c*) continuously monitor host (*H*) nests, trying to find chances for replacing eggs. Their egg‐laying capacity, as well as the host pair's antilaying response, are subject to reinforcement at the end of this stage. (b) and (c) show host behaviors. Nonoccupied hosts (b) hatch their own eggs. (c) Hosts detect parasite egg color and/or shape to choose to either reject or accept, and then hatch the remaining eggs. The white triangle is host nestlings, while the shaded one is cuckoo nestlings. Uppercase letters corresponded to different actions: *L*, ¬L, *D*, *A*, *U*, and *H* means laying, not laying, detected, accepted, unfertilized, and hatched, respectively. Denotations for the probability and reinforcement variables are seen in Table S1.

For the egg parasitizing processes, we defined two variables: First, the probability of successfully laying eggs in a host nest ($p_{f_{c},l}$) describes the female cuckoo's tactics, such as finding a suitable host nest by monitoring a potential host bird (Marton et al. 2019) and mimicking the appearance (Welbergen and Davies 2011) and voice (York and Davies 2017) of host predators. Second, the probability of preventing eggs from being replaced by foreign eggs in host nests ($p_{P_{h},l}$) reflects the capacity of hosts staying at the nest long enough to prevent parasitizing. The net probability of a female cuckoo ($f_{c}$) successfully parasitizing a host nest ($n_{h}$) and laying eggs (*L*) was calculated by subtracting $p_{f_{c},l}$ from $p_{P_{h},l}$ (Figure 3a). Notably, each female cuckoo ($f_{c}$) was assigned a preferred host species at model initialization, and this preference was inheritable. If all nests of the preferred host species were already parasitized, the model triggered a “back‐up” behavior, whereby the female cuckoo laid her egg in a randomly selected nest from an alternative host species (Sorenson et al. 2003). Detailed procedures for egg parasitism are described in Algorithm S7 in Appendix S1.

### 2.4 Egg Cheating and Detection Processes

The cheating and detection processes were modeled from two perspectives: egg color and egg shape. Because the success of parasite egg detection depends on both the similarity of cuckoo eggs to host eggs and the host's ability to discriminate parasitic eggs, we introduced paired probability measures—one representing the cuckoo's ability to mimic and one representing the host's ability to detect. For egg color, $p_{e_{c},c}$ denotes the probability that a cuckoo egg successfully mimics host egg color, while $p_{P_{h},c}$ represents the host pair's ability to detect color discrepancies. The net probability of successful detection by the host based on egg color was defined as $\max\left\{0,p_{P_{h},c}-p_{e_{c},c}\right\}$, allowing for stochastic variability on both sides. Similarly, for egg shape, $p_{e_{c},s}$ and $p_{P_{h},s}$ denote the cuckoo's mimicry ability and the host's detection ability, respectively. These paired probabilities were used to evaluate the overall likelihood of detecting and rejecting a parasite egg (*D*), denoted as $p_{h,d}$ (Equation 1). If detection fails, the host pair accepts the parasitic egg, resulting in successful parasitism (*A*).

(1) $$p_{h,d}=1-\left(1-\max\left\{0,p_{P_{h},c}-p_{e_{c},c}\right\}\right)\left(1-\max\left\{0,p_{P_{h},s}-p_{e_{c},s}\right\}\right)$$

Detailed procedures for egg cheating and detection based on color and shape are described in Algorithm S8 in Appendix S1.

### 2.5 Egg Incubating and Rearing Processes

**FIGURE 4:**
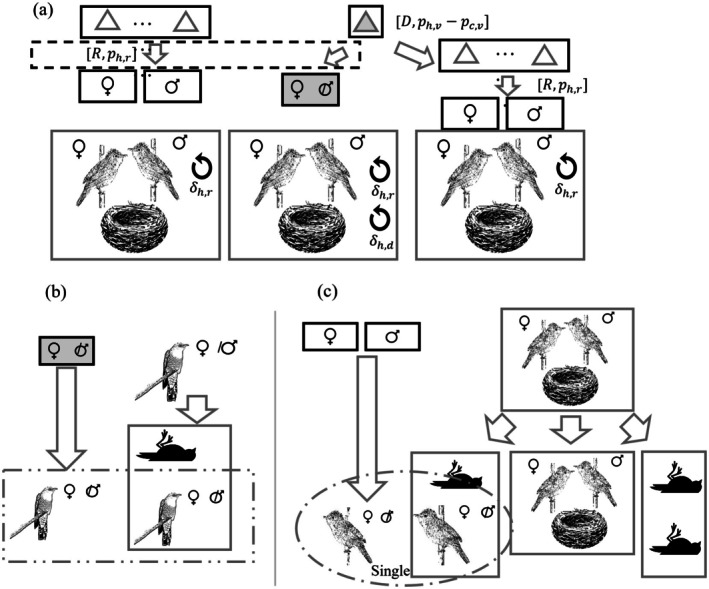
Simulations for the rearing phase and group updates. (a) The last opportunity for the host family to reject a parasite egg based on a vocal password. If still not detected, the egg hatches and the chick is raised to adulthood. After that, the groups, cuckoos (b) and hosts (c), update. Dead individuals are removed, and hatch‐year individuals are added to the model. Uppercase letters corresponded to an action: *R* means reared, D means detected. Denotations for the probability and reinforcement variables are seen in Table S1.

The first step in the incubation and rearing process involved removing unfertilized eggs (*U*). To account for fertilization success, we introduced two parameters: $p_{c,h}$ and $p_{h,h}$, representing the probabilities of cuckoo and host eggs being successfully fertilized, respectively. Only fertilized eggs were allowed to proceed to the next developmental stage. Depending on whether parasitism and fertilization were successful, the host nest could fall into one of four states prior to hatching: (1) nonparasitized (Figure 3b), (2) parasitized but the cuckoo egg was detected and removed, (3) parasitized but the cuckoo egg was unfertilized, or (4) parasitized with a fertilized cuckoo egg that successfully hatches (Figure 3c). In Scenario (4), the final opportunity for host parents to identify parasitic offspring occurs posthatching through recognition of species‐specific vocal passwords—unique acoustic signatures shared between host parents and their chicks (Colombelli‐Négrel et al. 2012). The probability of rejecting cuckoo chicks based on vocal mismatch was modeled as the difference, $p_{P_{h},v}-p_{e_{c},v}$, following the same paired probabilistic framework used in earlier detection stages (Figure 4a). If parasitism was detected (D), the host pair abandoned the nest—including both eggs and nestlings—rebuilt a new nest, and restarted the reproductive cycle following the same mating and egg generation procedures. Followed by above steps, the host parents would rear offsprings regardless of species (Dawkins and Krebs 1979; Heeb et al. 2003; Caro et al. 2016), and if the hosts failed to reject foreign eggs, any hatched parasitic chick would kill the host's biological nestlings and dominate the brood (Broom et al. 2008). The overall probability of successfully rearing nestlings, $p_{h,r}$, was defined as the ratio of chicks raised to adulthood. To account for resource limitations, a population‐regulating coefficient was applied to $p_{h,r}$, decaying toward zero as the population approached its carrying capacity, consistent with logistic growth dynamics. Details about egg incubating and rearing processes were listed in Algorithm S9 in Appendix S1.

### 2.6 Reinforcement and Group Updates

To model reinforcement dynamics, we defined reinforcement as a small, noninheritable increase in specific behavioral probabilities, applied only to individuals that directly experienced relevant interactions. In other words, reinforcement effects were individual‐specific and not transmitted to offspring; each offspring needed to independently undergo the same experiences to acquire reinforcement. When reinforcement was triggered, the affected individual's probability for the following year ($p_{t+1}$) was updated by an increment δ, as shown in Equation (2):

(2) $$p_{t+1}=p_{t}+\delta$$

During the processes from mating to rearing, the following reinforcement mechanisms were implemented: $\delta_{c,l}$, representing the reinforcement of cuckoo egg‐laying success, and $\delta_{h,l}$ representing reinforcement of the host's nest‐guarding ability. Host detection capabilities were further reinforced through $\delta_{h,d}$, composed of three components—$\delta_{P_{h},c}$, $\delta_{P_{h},s}$, and $\delta_{P_{h},v}$—which corresponded to enhanced detection based on egg color, egg shape, and chick vocalization, respectively. These were applied if the host pair was deceived and reared nonhost offspring during the current year. Additionally, $\delta_{h,r}$ represented reinforcement gained through successful rearing experience. At the end of each simulation cycle, individuals that had reached the end of their lifespan were removed. If only one member of a host pair died, the surviving individual sought a new partner in the following year to form a new reproductive unit (Figure 4b,c and Algorithms S9 and S10 in Appendix S1).

### 2.7 Model Validation and Simulation

We validated the model by comparing its outputs to field observation data under identical conditions (Brooke et al. 1998; Samaš et al. 2016). Initial parameter values for the baseline (year zero) settings—including the maximum and average lifespans of cuckoos and hosts, the maximum and average number of eggs produced, and the success rates for egg laying, parasitism detection, fertilization, and chick rearing—were derived from published studies and avian databases (Robinson 2005; Erritzøe et al. 2012). Where empirical data were unavailable, such as probability parameters related to paired behaviors (e.g., egg laying vs. antilaying, and egg deception vs. detection) were estimated based on the authors' best estimates. To incorporate stochastic variability while enabling gradual behavioral shifts, standard deviations for each probability were set at two orders of magnitude below their respective means. Similarly, for probabilities subject to reinforcement, the reinforcement factor was also set at two orders of magnitude below the corresponding mean value. The model was run over the same number of years as observed in the field, and the population information at each year was recorded. In order to keep consistent with field observations, we compared the parasitism ratio (PR), which is the rate of observed parasitized nests over the total number of observed nests, between our simulation results and field observation data.

Following validation, we conducted simulations to investigate cuckoo population dynamics, the potential for “back‐up” behavior, and the adaptable recognition–rejection (RR) range. RR is defined as the host pair's capacity to detect cuckoo eggs based on color or shape, minus the cuckoo's corresponding capacity to mimic these traits. For all host populations, we standardized the mean values of $p_{h,c}$ and $p_{h,s}$ to 40%. To assess the influence of initial conditions, we created five virtual cuckoo populations (A–E), each defined by distinct combinations of egg color and shape cheating probabilities. Population A was assigned with medium values for both color and shape (15% $p_{e_{c},c}$ and 15% $p_{e_{c},s}$, corresponding to 25% color RR and 25% shape RR). Population B had high color but low shape RR (10% $p_{e_{c},c}$ and 20% $p_{e_{c},s}$, or 30% color RR and 20% shape RR), with a maximum lifespan of 7 years. Population C had low color but high shape RR (20% $p_{e_{c},c}$ and 10% $p_{e_{c},s}$, or 20% color RR and 30% shape RR), with a maximum lifespan of 10 years. Population D was assigned with extremely high RR for both color and shape (10% $p_{e_{c},c}$ and 10% $p_{e_{c},s}$, or 30% color RR and 30% shape RR). Population E had extremely low RR values for both (25% $p_{e_{c},c}$ and 25% $p_{e_{c},s}$, or 15% color RR and 15% shape RR). These populations were initially placed together in unfragmented habitats (baseline conditions) and simulated over 300 years with 100 runs. To account for potential discrepancies in absolute population counts, we used relative population (RP), defined as the ratio of each year's population to the year‐zero population, as the metric for comparison among the population dynamics of cuckoo population A to E.

To evaluate the role of “back‐up” behavior, we repeated these simulations with identical settings but without enabling “back‐up” responses in cuckoos, allowing us to estimate the behavioral potential of this strategy. We then systematically explored all possible combinations of color and shape RR values—ranging from their respective minimum to maximum values in 5% increments—to identify the adaptable RR range that supports long‐term cuckoo survival. To isolate the effects of RR traits without competitive interactions between cuckoo populations, we simulated only one cuckoo group at a time while keeping all other settings fixed as in population A. This setup enabled a focused investigation of the coevolutionary dynamics between a single cuckoo population and its host populations. After completing simulations under unfragmented conditions, we introduced HLF by reducing host environmental capacity to 75% (moderate HLF) and 50% (severe HLF) of baseline levels. Correspondingly, the year‐zero population sizes of both cuckoos and hosts were proportionally scaled down. All other model parameters were held constant. The full suite of simulations was then repeated under these HLF conditions to assess changes in population trends, behavioral potential, and RR adaptability in response to environmental stress.

## 3 Results

### 3.1 Model Validation Performance

**FIGURE 5:**
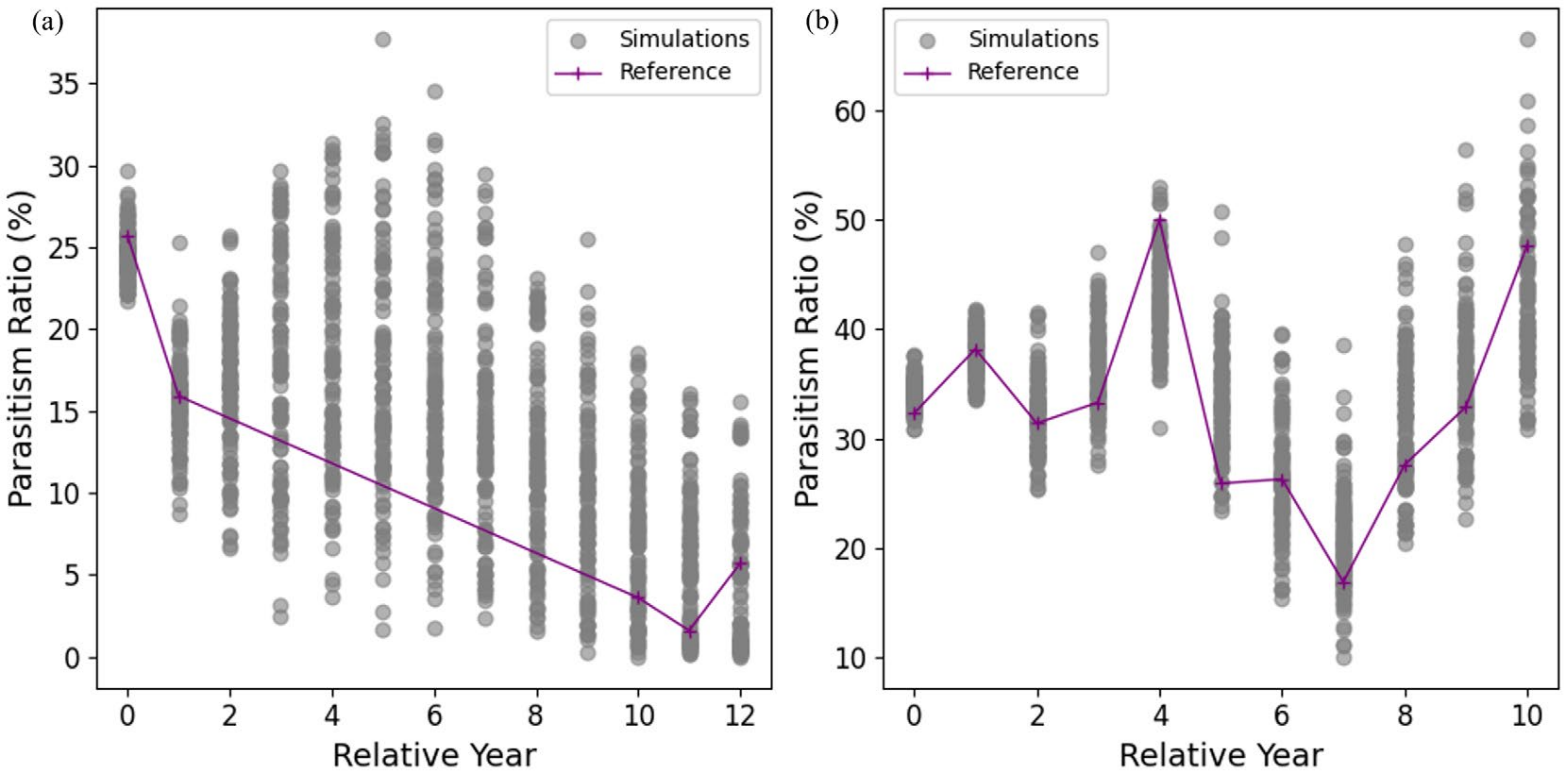
Model validation with field observation data. Field data for (a) and (b) were extracted from Brooke et al. (1998) and Samaš et al. (2016), respectively. In each plot, gray dots depict model sampling results and purple lines indicate field observations.

The validation results are presented in Figure 5. Gray dots represent the parasitism ratio from individual simulation runs, while the purple line denotes field observation data. For the first field dataset (Brooke et al. 1998), all four observations after year 0 (i.e., Years 1, 10, 11, and 12) fall within the range of simulation outputs. The final‐year observed parasitism rate is 5.7%, corresponding to the 42nd percentile of the simulated values, with a simulation median of 7.2%. For the second dataset (Samaš et al. 2016), despite high variability in the field measurements, all post‐year‐0 observations are within the simulated range. In the final year, the observed value is 47.7%, corresponding to the 78th percentile, while the simulation median is 40.7%. These results indicate that the model effectively captures the observed range and distribution of parasitism ratios, providing support for its validity in reproducing field‐measured dynamics.

### 3.2 Cuckoo Population Dynamics

**FIGURE 6:**
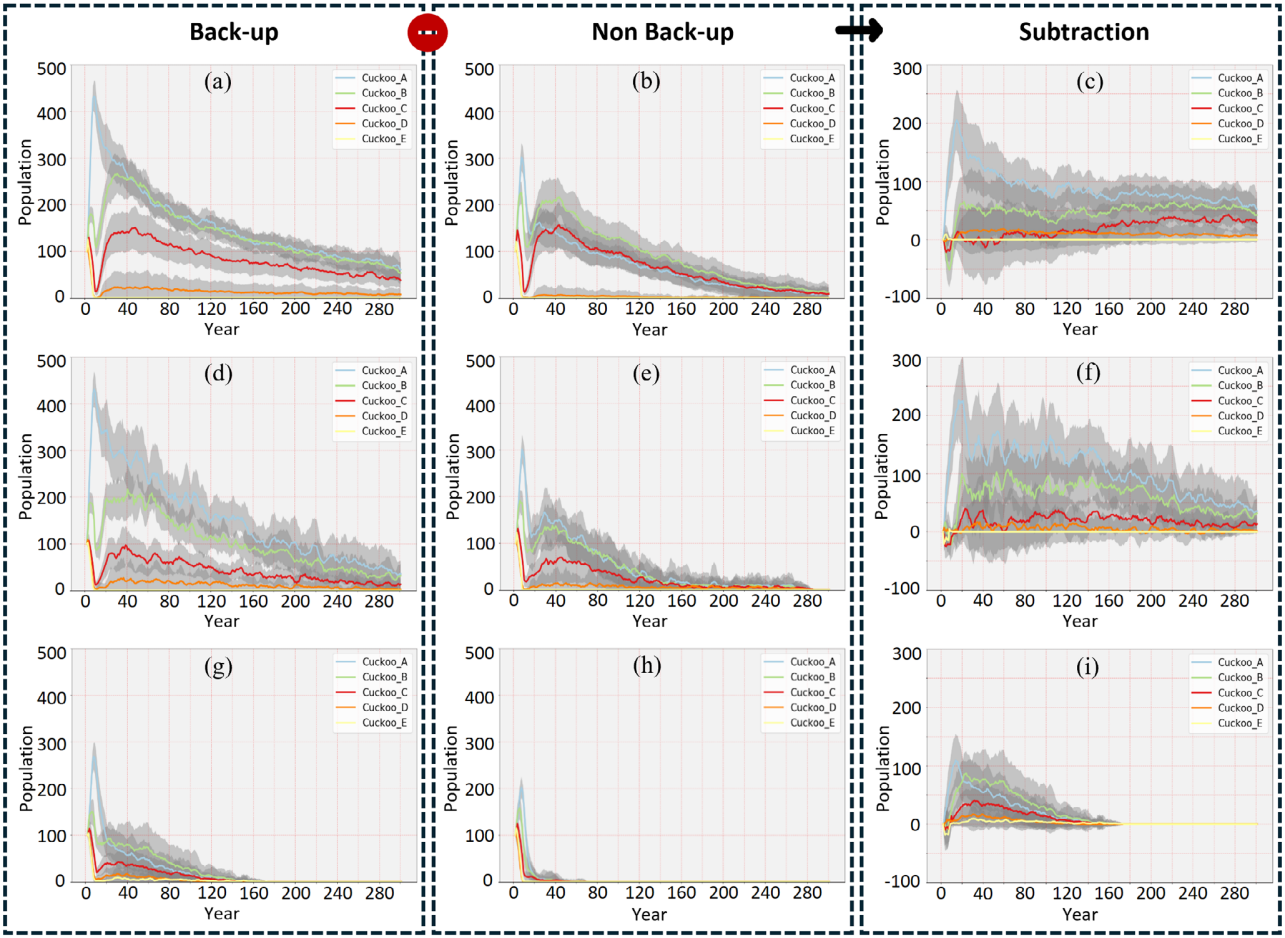
Yearly comparison of virtual common cuckoo relative population (RP) sizes, with and without “backup” behaviors, in unfragmented (a–c), moderate HLF (d–f), and severe HLF (g–i) conditions. The first column depicts the cuckoo RP fluctuation with a “backing‐up” strategy while the second column shows cuckoo RP fluctuation without that strategy. Sampling variances are shown as shaded areas around every solid line. Cuckoo_A, medium rejection rate (RR) based on egg color and shape; Cuckoo_B, high shape and low color RR; Cuckoo_C, low shape and low color RR; Cuckoo_D, extremely high color and shape RRs; Cuckoo_E, extremely low shape and color RRs.

In unfragmented habitats (baseline), the five simulated cuckoo populations exhibited four distinct types of long‐term trends based on their initial recognition–rejection (RR) values, as measured by the mean annual relative population (RP) over a 300‐year simulation period (Figure 6a). Population E, characterized by extremely low RR values for both egg color and shape, experienced a rapid decline, reaching extinction within 20 years. This result suggests that excessive cheating ability significantly reduces host reproductive success, leading to a collapse in the host population and, consequently, causing the extinction of cuckoos. Population A, with medium RR values for both traits, showed an initial sharp increase—reaching approximately 450% of its starting population—followed by a gradual decline and stabilization around 60% of the initial population size by year 300, indicating that moderate cheating ability yields the highest long‐term survival potential. Populations B and C, which each had one high and one low RR trait (high color and low shape in B; high shape and low color in C), showed fluctuating dynamics in the early years, then rebounded and stabilized at lower equilibrium levels—approximately 55% for B and 40% for C. These outcomes suggest that cuckoo populations with asymmetrical RR values can persist but face higher uncertainty and more volatile trajectories. Notably, population B achieved a slightly higher RP than C, which may reflect the impact of lifespan differences: the longer lifespan assigned to population C potentially enhances parasitism success via reinforcement over time but also imposes greater pressure on host populations, which may feedback to reduce cuckoo abundance. Lastly, population D, with extremely higher RR values, experienced an initial sharp drop, followed by a slight rebound and long‐term persistence at a low RP of 9%, suggesting that minimal cheating capacity, while insufficient for dominance, may still support low‐level survival when host pressure is reduced. Together, these patterns illustrate the importance of initial RR traits in shaping long‐term cuckoo population dynamics under stable environmental conditions.

### 3.3 Potential of Back‐Up Behavior

To evaluate the long‐term potential of the “back‐up” behavior, we quantified the subtraction of relative population (SRP) for each group as the difference between the 300th‐year relative population (RP) with “back‐up” behavior enabled (Figure 6a) and the RP without it (Figure 6b). Simulations without “back‐up” behavior revealed that population E consistently declined to extinction, mirroring earlier results, while the remaining populations (A–D) exhibited fluctuations during the initial 30 years, followed by a relatively sharp decline to extremely low RP values by Year 300 (Figure 6b). The resulting SRP values—0% for E, 9% for D, 29% for C, 42% for B, and 51% for A (Figure 6c)—highlight considerable variation in behavioral potential across groups. Population A, with moderate RR values for both egg color and shape (~25%), demonstrated the highest behavioral potential, with its population at Year 300 increased by 51% of its initial size due to the presence of “back‐up” behavior. Populations B and C also showed substantial gains (42% and 29%, respectively), suggesting that “back‐up” behavior can effectively enhance long‐term survival even when RR traits are asymmetrical. In contrast, the low SRP in population D and the null SRP in population E indicate that when RR values are either too low or too high, “back‐up” behavior alone is insufficient to support long‐term survival, likely due to fundamental constraints in parasitism success or excessive pressure on host populations.

### 3.4 Impact of HLF on Population Dynamics and Behavior Potential

The effects of moderate (25% environmental capacity loss) and severe (50% environmental capacity loss) HLF on cuckoo population dynamics are shown in Figure 6d,g, respectively. Under moderate HLF, population D went extinct by Year 300, while populations A, B, and C survived but exhibited reduced equilibrium relative populations (RP). Specifically, populations A and B declined to approximately 30% RP, and population C declined to approximately 10% RP. Under severe HLF, all cuckoo populations collapsed to extinction by year 300, indicating that no population could withstand the compounded pressures of intense habitat loss. Regarding the behavioral potential of “back‐up” strategies, under moderate HLF (Figure 6d–f), SRP values for populations A, B, and C decreased to 33%, 29%, and 12%, while population D's SRP dropped to 0% due to extinction prior to Year 300, even with “back‐up” behavior enabled. Population E also maintained a 0% SRP, reflecting consistent extinction across all scenarios. These findings suggest that while moderate HLF reduced behavioral potential across all groups, Population A (medium RR settings) still maintained the highest potential. Under severe HLF (Figure 6g–i), no population maintained sufficient size or persistence time throughout the 300‐year simulation period, regardless of whether the “back‐up” mechanism was available. This indicates that once HLF surpasses a certain threshold, the coevolutionary process becomes highly unstable, and “back‐up” behavior alone may be insufficient to re‐establish equilibrium, ultimately leading to extinction (Figure 6g–i).

### 3.5 Impact of HLF on RR Equilibrium

**FIGURE 7:**
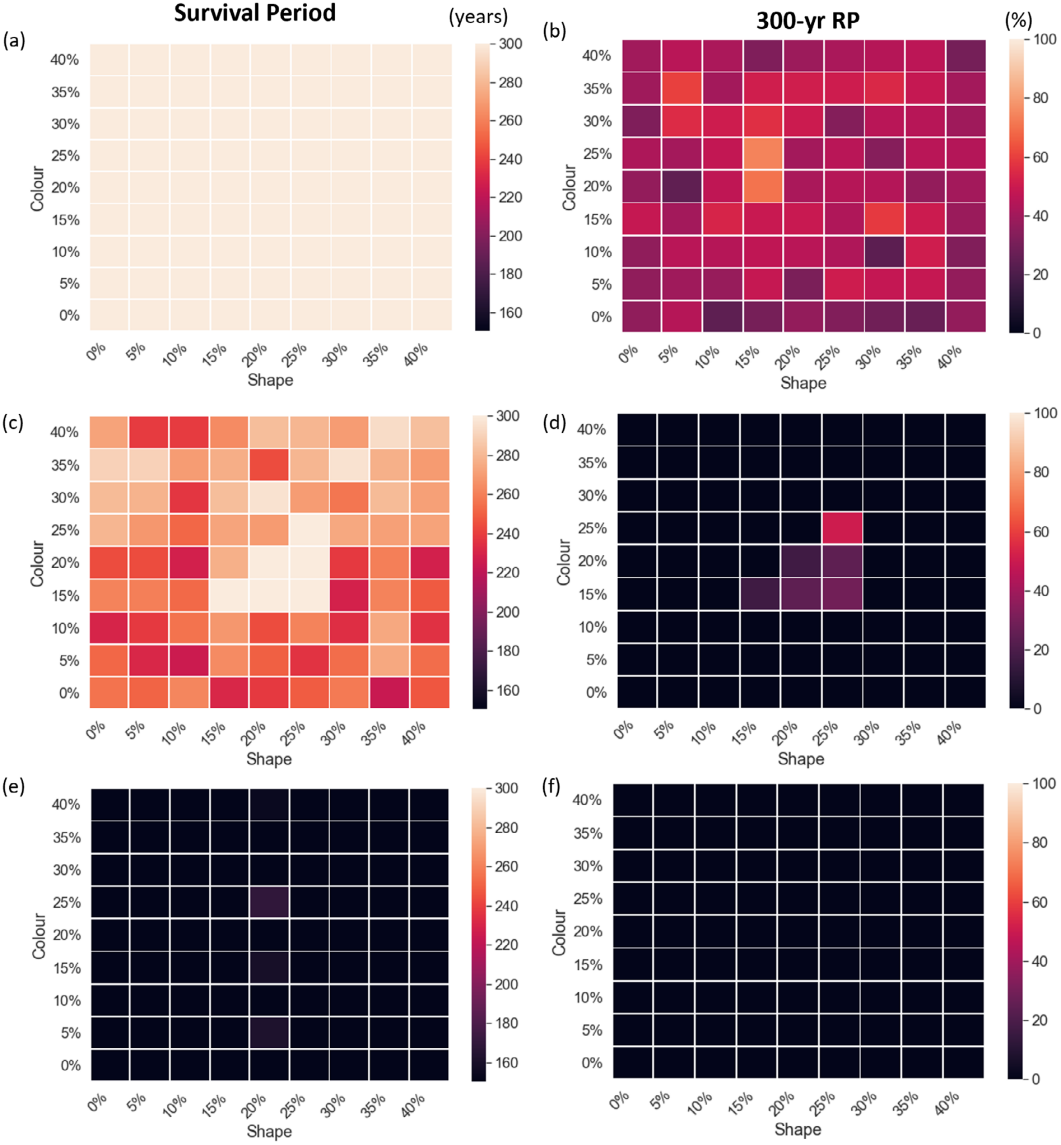
Joint effects of different initial shape and color rejection rates (RRs), with and without habitat loss and fragmentation (HLF), on cuckoo population dynamics. Simulations in an unfragmented habitat (a and b), under moderate HLF (c and d), and under severe HLF (e and f). Given the various RRs, the left column depicts the survival periods of the simulated cuckoo groups, and the right column shows the cuckoo groups' 300‐year relative population (300‐year RP) fluctuations.

To assess how habitat loss and fragmentation (HLF) influence the recognition–rejection (RR) trait range conducive to long‐term cuckoo survival, two metrics were used across all RR configurations: (1) survival period, defined as the number of years a population persisted before extinction, and (2) 300‐year relative population (300‐year RP), representing the population size at the end of the simulation period. The results under unfragmented (baseline) habitat conditions are presented in Figure 7a,b. In this scenario, cuckoo populations with all RR combinations were able to survive the full 300‐year simulation period; however, populations with medium RR values (ranging from 15% to 25% for both color and shape) maintained relatively high 300‐year RP values, exceeding 40%. In contrast, populations with either extremely low (0%) or extremely high (40%) RR values for either color or shape exhibited substantially lower 300‐year RP values, indicating suboptimal long‐term stability. Under the moderate HLF scenario (Figure 7c,d), most RR combinations resulted in cuckoo extinction before Year 260, and only a narrow subset of RR settings enabled persistence. Notably, populations with intermediate RR values (specifically, 25% for both color and shape) demonstrated greater resilience and maintained 300‐year RP values above 40%, consistent with their performance in the baseline scenario. This suggests that moderate RR traits are more adaptable under moderate HLF conditions. However, even these resilient configurations showed reduced 300‐year RP compared to the unfragmented scenario, highlighting the pressure of reduced environmental capacity under moderate HLF on cuckoo groups. In the severe HLF scenario (Figure 7e,f), extinction risk increased dramatically. All cuckoo populations, regardless of RR settings, experienced shorter survival periods, with most going extinct around Year 175. No group sustained a viable population through the full 300 years, and even the most adaptable RR settings failed to preserve long‐term persistence. These results suggest that as habitat conditions deteriorate, the spectrum of RR values compatible with long‐term survival becomes increasingly narrow—while moderate HLF imposes selective pressure that narrows the adaptive window, severe HLF greatly accelerates extinction risk.

## 4 Discussion

### 4.1 Model's Innovations and Limitations

Our simulation model operates at the individual level, capturing trait variation, inheritance, and behavioral responses, while allowing outcomes to be aggregated and analyzed at the population level. This structure provides new insights into understanding the coevolutionary interactions of cuckoos and their hosts under varying degrees of HLF. In field observations, adequate sample size is often a key limiting factor, such as those used in coevolutionary studies (Brooke et al. 1998; Lindholm 1999; Lahti 2005; Thomson et al. 2016). However, computer simulation provides us with an alternative and powerful tool to overcome those observational limits, as well as bias from spatial heterogeneity, sampling efforts, and duration.

Most of the previous models exploring avian brood parasitism addressed either individual or population‐level dynamics but did not integrate the two. Models focusing on the population level are usually built on a series of empirical statistical formulas that often ignore individual variation and inter‐ and intraspecific interaction details (Ducatez 2014; Antonson et al. 2020). The parameters of those models are usually site‐specific and may be unsuitable in other settings. On the other hand, several previous models (Lindholm 1999; Harrison and Broom 2009; Hauber 2014) approached questions by producing individual‐level simulations that incorporate a series of partial differential and/or probability‐based equations that both construct coevolutionary progress and generate optimal solutions. Such simulations are designed to provide the minimum cost and/or maximum profit to cuckoos and/or their hosts. To achieve optimum solutions, most of those models assume that all individuals rationally select the most beneficial options in all periods (Skubic et al. 2004; Hauber 2014), a process not grounded in the reality that inheritable behavioral changes occur completely randomly, and individuals with more adaptable behaviors are chosen through natural selection to survive.

Unlike both the previous models and traditional field studies, our model simulates coevolutionary progress by stochastically including all individuals, a process that overcomes field‐based sampling constraints. Also, stochastic sampling randomly assigns individual variable values within a population and randomly passes those values to the next generation, rather than generating equations or formulas that may have bias caused by inaccurate estimation of the equations' or formulas' parameters on the results. In contrast, the reinforcement part of our model separates learned from inheritable effects, which makes the model more comparable to the real ecological process. The approaches we developed in this study also have potential use in future coevolutionary studies because, with slight parameter modifications, they can be fitted to investigate other coevolutionary ecological processes.

While our model focuses primarily on the negative impacts of HLF—represented as reductions in environmental carrying capacity—we acknowledge that habitat fragmentation can also produce complex, and in some cases beneficial, effects on population dynamics and coevolutionary processes (Fahrig 2003; Valerio et al. 2016). For example, in certain systems, fragmented landscapes may enhance biodiversity and create new ecological opportunities for coevolution (Fontúrbel and Murúa 2014; Fahrig et al. 2019). For some bird species, the effects of HLF on species richness or population viability may depend not only on the degree of HLF but also on the total amount of available habitat (Smith et al. 2011; De Camargo et al. 2018). These nuances are not captured in the current model but represent promising avenues for future development. Moreover, HLF is not solely characterized by reductions in habitat amount. In reality, fragmentation often produces spatially discrete habitat patches, but movement between them is not inherently restricted; in fact, fragmentation per se can in some cases facilitate between‐patch movement, increase habitat heterogeneity, and enhance landscape complementation, depending on species‐specific traits and the broader spatial context (Fahrig et al. 2019). A more ecologically realistic simulation framework would represent fragmented environments as networks of habitat patches, with interpatch distances influencing dispersal, connectivity, and gene flow. Incorporating such patch‐based spatial structure into future iterations of the model would allow for exploration of metapopulation dynamics, source–sink relationships, and the tradeoffs between isolation and connectivity under different HLF scenarios.

Although the anthropogenic activities related to HLF are always referred to human land use, mining, and urbanization, the macroecological changes (e.g., climate change) that are at least partially caused by humans should not be escaped from our sights (Coristine and Kerr 2011; Scanes 2018). In this regard, the habitat conditions may fluctuate all the time, making some animal species unable to catch up under the joint impacts from anthropogenic climate changes (Whiteman et al. 2015; Bay et al. 2018). The increased uncertainty and fluctuation of the habitat, such as food availability, seasonality, and extreme weather events, associated with such large‐scale changes should be considered in future modeling and studies.

In addition, while our model integrates stochasticity and reinforcement to simulate key behavioral and evolutionary mechanisms, we acknowledge that additional ecological and behavioral dynamics—such as behavioral plasticity, social learning, cultural transmission, and interspecific interactions beyond parasitism—may also play critical roles in shaping coevolutionary outcomes and fine‐scale variation in coevolutionary trajectories (Davies and Welbergen 2009; Feeney and Langmore 2013; Thorogood and Davies 2013). These complex processes remain difficult to quantify and were not explicitly included in the current framework. However, future work that integrates empirical datasets and behavioral experiments may help refine these mechanisms within simulation models and improve our understanding of how animal behavior and coevolution respond to rapidly changing ecological conditions.

### 4.2 Sensitivity Analysis

**FIGURE 8:**
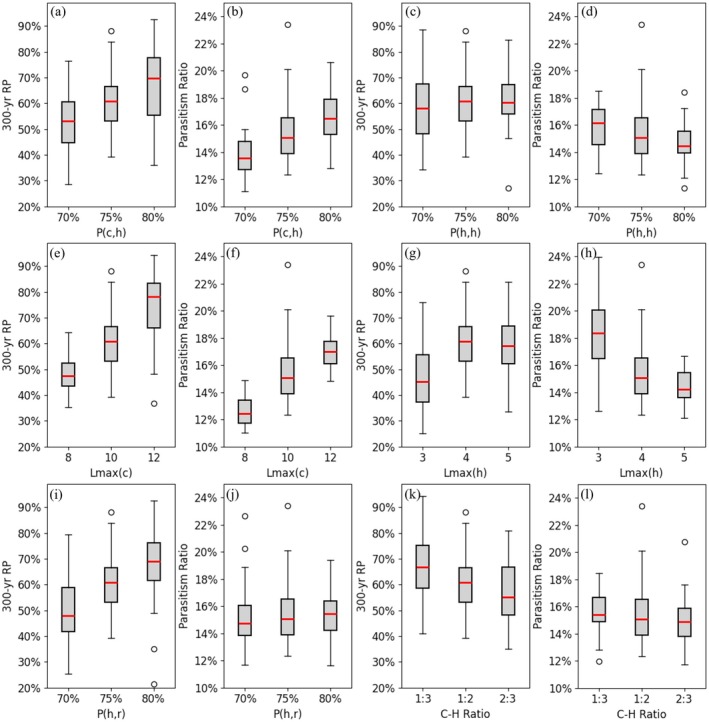
Sensitivity test of model input parameters. Panels (a, b) show simulation results for the 300‐year relative population (RP) and parasitism ratio under varying cuckoo egg fertilization rates; (c, d) under varying host egg fertilization rates; (e, f) under varying cuckoo maximum lifespans; (g, h) under varying host maximum lifespans; (i, j) under varying host rearing rates; and (k, l) under varying initial cuckoo‐to‐host population ratios.

As a stochastic model, our simulation outputs naturally exhibited variability, and both the magnitude and distribution of results were influenced by initial model settings. To evaluate the sensitivity of the model to key biological and demographic parameters, we conducted a sensitivity analysis on the following inputs: the fertilization rate of cuckoo eggs ($p_{c,h}$), the fertilization rate of host eggs ($p_{h,h}$), the maximum lifespan of cuckoos ($L_{c,\max}$), the maximum lifespan of hosts ($L_{h,\max}$), the host's successful rearing rate ($p_{h,r}$), and the initial population size ratio between cuckoos and hosts (C–H ratio). For simplicity, the analysis was conducted on a single cuckoo group interacting coevolutionarily with its host population under baseline conditions corresponding to cuckoo A in an unfragmented habitat. Each parameter was varied at a lower and a higher setting around its baseline. The tested parameter ranges were: $p_{c,h}$ and $p_{h,h}$ at 70%, 75%, and 80%; $L_{c,\max}$ at 8, 10, and 12 years; $L_{h,\max}$ at 3, 4, and 5 years; $p_{h,r}$ at 70%, 75%, and 80%; and the C–H ratio at 1:3, 1:2, and 2:3. Under the baseline setting, the median relative population (RP) at year 300 was 60.3%, and the parasitism ratio (PR) was 15.1% (Figure 8). The sensitivity results (Figure 8a–l) indicate that RP is generally more responsive than PR to parameter changes. For instance, modifying $L_{c,\max}$ shifted the 300‐year RP from 47.5% (lower value) to 78.1% (higher value), while PR ranged from 12.4% to 17.0%. Changes in $L_{h,\max}$ similarly affected RP more strongly, from 45.2% to 59.2%, compared to a PR range of 14.2% to 18.4%. Varying $p_{c,h}$ also produced considerable differences in RP (53.1% to 69.7%) and moderate shifts in PR (13.6% to 16.5%), as well as $p_{h,r}$ for RP (47.9% to 67.9%) but not PR (14.8% to 15.4%). In contrast, parameters such as $p_{h,h}$ and the C–H ratio led to more modest changes in both RP and PR. Overall, these findings underscore that relative population is more sensitive to input variation than parasitism ratio, particularly for life‐history traits like lifespan and fertilization success. Consequently, to improve the reliability of long‐term projections using this model, it is especially important to obtain accurate field measurements of cuckoo and host lifespans, egg fertilization rates, and fledging success. Prioritizing these parameters in field studies will enhance model calibration and strengthen confidence in forecasting cuckoo group dynamics under various ecological conditions.

### 4.3 Behavioral Adaptation Processes of a Coevolutionary Species Pair Subjected to HLF

Adaptations to dynamic environments and interspecific interactions drive species evolution. In the Anthropocene, the rapid expansion of human land use has led to widespread habitat loss and fragmentation (HLF), posing significant challenges for wildlife survival (Barnosky et al. 2011; Ceballos et al. 2015). In addition to the direct harvesting and persecution that lead to catastrophic wildlife decline, anthropogenic activities profoundly influence the evolutionary progress of species living in human‐dominated landscapes (Crooks et al. 2017). One result of those activities, HLF, affects biodiversity by leading to population declines due to restricted animal movements and gene flow. But the question of to what extent HLF affects coevolutionary progress has been overlooked. Our model's results suggest that HLF could disrupt coevolution, and severe HLF may accelerate the extinction of parasitizing species.

How broadly HLF affects a species' behavioral adaptations depends on patch size and the species' responses to it. Also, patch size is one of the most important predictors used to assess local population extinction risks (Crooks et al. 2017). Since species inhabiting large areas tend to be habitat generalists (Brown 1984), parasitic species may inhabit even larger areas, thus allowing them to adopt new reproduction strategies to avoid a dramatic population decreases. Moreover, one theory suggests that avian interspecific brood parasitism is a facultative behavior that evolved from intraspecific brood parasitism, and thus it possesses high plasticity (Britton et al. 2007; Kilner and Langmore 2011). Therefore, most previous models may have underestimated that behavioral potential, as demonstrated by the “back‐up” behavior in our current model.

Additionally, the possible relationships and interactions between species are difficult to define and quantify. To evaluate the adaptive capacity of cuckoo populations under disturbance, we used Subtraction of Relative Population (SRP) values to assess their responses to both moderate and severe habitat loss and fragmentation (HLF). While our model incorporates individual‐level behavioral variation and coevolutionary processes, it does not simulate a gradual onset of HLF. Instead, for simplicity, environmental capacity is reduced abruptly at year zero, representing an immediate transition to fragmented conditions. As a result, the model does not account for the progressive accumulation of behavioral adaptations in response to habitat deterioration over time, a process previously documented in behaviors such as dispersal, food searching, and foraging (Cattarino et al. 2016; Cornelius et al. 2017). Despite this limitation, our results still supported that under the positive selective pressure caused by HLF, most particularly severe HLF, the benefits of a parasite adjusting its behavior to find a new host species are not enough to avoid its extinction. Subsequent models should incorporate as many of those fluctuations as possible, as well as the behavioral accumulation process just mentioned, and then compare those results with ours. Such additions may provide more accurate and dynamic results.

More importantly, the essence of HLF's effect on population dynamics is the fluctuation of genomic frequencies and resource availability in a given system (Keyghobadi 2007), which has always been neglected. If genomic data of two involving parties are available (Hu et al. 2021), for example, by resequencing adequate amounts of individuals, and could be incorporated into the stochastic process, we may have more advanced and clear insights into the mechanisms driving the coevolutionary process. How resource availability influences genomic drifting of two coevolutionary competitors, and within this race, how one party's genomic variation affects the other party's, are all fascinating questions. Such studies on to what extent the HLF can influence the genomic frequencies within coevolutionary populations are further needed.

This study employs stochastic and reinforcement simulation while considering different HLF scenarios and is the first both to provide insights into the effects that HLF have on coevolutionary progress and to quantify a species' responses to such disturbances. Future modeling studies may consider incorporating more parameters and detailed settings, such as varied patch size, distance, habitat quality, edge effect, and the behavioral learning process in host, etc., into the simulation and improve the model accuracy and robustness with more empirical data. These results will improve our understanding of the ecological consequences of human‐mediated HLF, and raise awareness among habitat managers to guide future conservation efforts accordingly.

## Author Contributions

**Wei Wang:** conceptualization (equal), data curation (equal), formal analysis (lead), methodology (equal), software (lead), validation (lead), visualization (equal), writing – original draft (equal), writing – review and editing (equal). **Timothy Van Deelen:** conceptualization (supporting), writing – review and editing (equal). **Fuwen Wei:** conceptualization (supporting), supervision (supporting). **Sheng Li:** conceptualization (equal), writing – review and editing (equal). **Luping Wang:** conceptualization (equal), formal analysis (equal), methodology (equal), validation (equal), visualization (equal), writing – original draft (equal), writing – review and editing (equal).

## Conflicts of Interest

The authors declare no conflicts of interest.

## Supporting information


Appendix S1.

Appendix S2.

## Data Availability

The Python script, parameter settings, and tutorials are publicly available at: https://github.com/wwang487/CuckooData.git.
