# Supplementary material for: Anthropogenic Habitat Loss and Fragmentation May Alter Coevolutionary Progress as Examined in a Brood Parasitism Model
Source: Ecol Evol. 2025 Jul 16;15(7):e71721. doi: 10.1002/ece3.71721 (PMC12264393; doi:10.1002/ece3.71721)

**Table .1: Meaning of Mathematical Indices**

| **Variable Name** | **Description** |
| --- | --- |
| *pc,l* | The probability of cuckoos’ egg to be successfully laid |
| *pc,c* | The cuckoo’s color cheating factor |
| *pc,s* | The cuckoo’s shape cheating factor |
| *pc,v* | The cuckoo’s vocal password cheating factor |
| *pc,h* | The probability of cuckoos’ egg to be successfully fertilized |
| *ph,l* | The host’s anti-laying factor |
| *ph,c* | The host’s base cheating detection probability based on color |
| *ph,s* | The host’s base cheating detection probability based on shape |
| *ph,v* | The host’s base cheating detection probability based on vocal password |
| *ph,h* | The probability of hosts’ egg to be successfully fertilized |
| *ph,r* | The probability of host to successfully hatch and breed an egg |
| *δp_c,l_* | The yearly learning reinforcement factor of *p_c,l_* |
| *δp_h,l_* | The yearly learning reinforcement factor of *p_h,l_* |
| *δph,c* | The yearly learning reinforcement factor of *p_h,c_* |
| *δph,s* | The yearly learning reinforcement factor of *p_h,s_* |
| *δph,v* | The yearly learning reinforcement factor of *p_h,v_* |
| *δph,r* | The yearly learning reinforcement factor of *p_h,r_* |
| *Nc,e* | Number of cuckoo eggs per female cuckoo |
| *N_h_* | Number of potential host species |
| *Nc,P* | Cuckoo group population |
| *Nh,e* | Number of host eggs per host family |
| *Nh,P* | Host group population |
| *Nh,P*0 | Host environmental capacity |
| *L_c_* | Cuckoo lifetime |
| *L_h_* | Host lifetime |
| *A_c_* | Cuckoo age |
| *A_h_* | Host age |
| *S_c_* | Cuckoo species name |
| *S_h_* | Host species name |
| *C* | List of cuckoo species names |
| *H* | List of host species names |
| C | Set of all cuckoo groups |
| H | Set of all host groups |
| U | Set of all host unpaired groups |
| C*S_c_* | The cuckoo group of species *S_c_* |
| H*S_h_* | The host group of species *S_h_* |
| U*S_h_* | The Unpaired host group of species *S_h_* |
| *F_c_* | Set of Female cuckoo group |
| *f_c_* | An individual female cuckoo |
| *M_c_* | Set of Male cuckoo group |
| *m_c_* | An individual male cuckoo |
| *F_h_* | Set of Female host group |
| *f_h_* | An individual female host |
| *M_h_* | Set of Male host group |
| *m_h_* | An individual male host |
| *P_h_* | Set of host pairs |
| {*f_h_,m_h_*} | A host pair |
| *n*{*f_h_,m_h_*} | Nest of host pair {*f_h_,m_h_*} |
| *p_g_* | Probability coefficient to determine gender |
| *p_d_* | Probability of successfully detecting the cheating |
| *κ_h_* | Logistic breeding factor related with population density |
| *σc,i* | Variation coefficient for cuckoo values (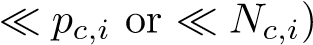 |
| *σh,i* | Variation coefficient for host values (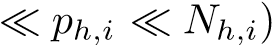 |
| *kc,i* | Variation coefficient for cuckoo discrete integral values |
| *kh,i* | Variation coefficient for host discrete integral values |
| *λc,i* | Variation coefficient for cuckoo discrete integral values |
| *λh,i* | Variation coefficient for host discrete integral values |
| *λe_c_,i* | Poisson parameter for cuckoo eggs |
| *λe_h_,i* | Poisson parameter for host eggs |

**Algorithms**


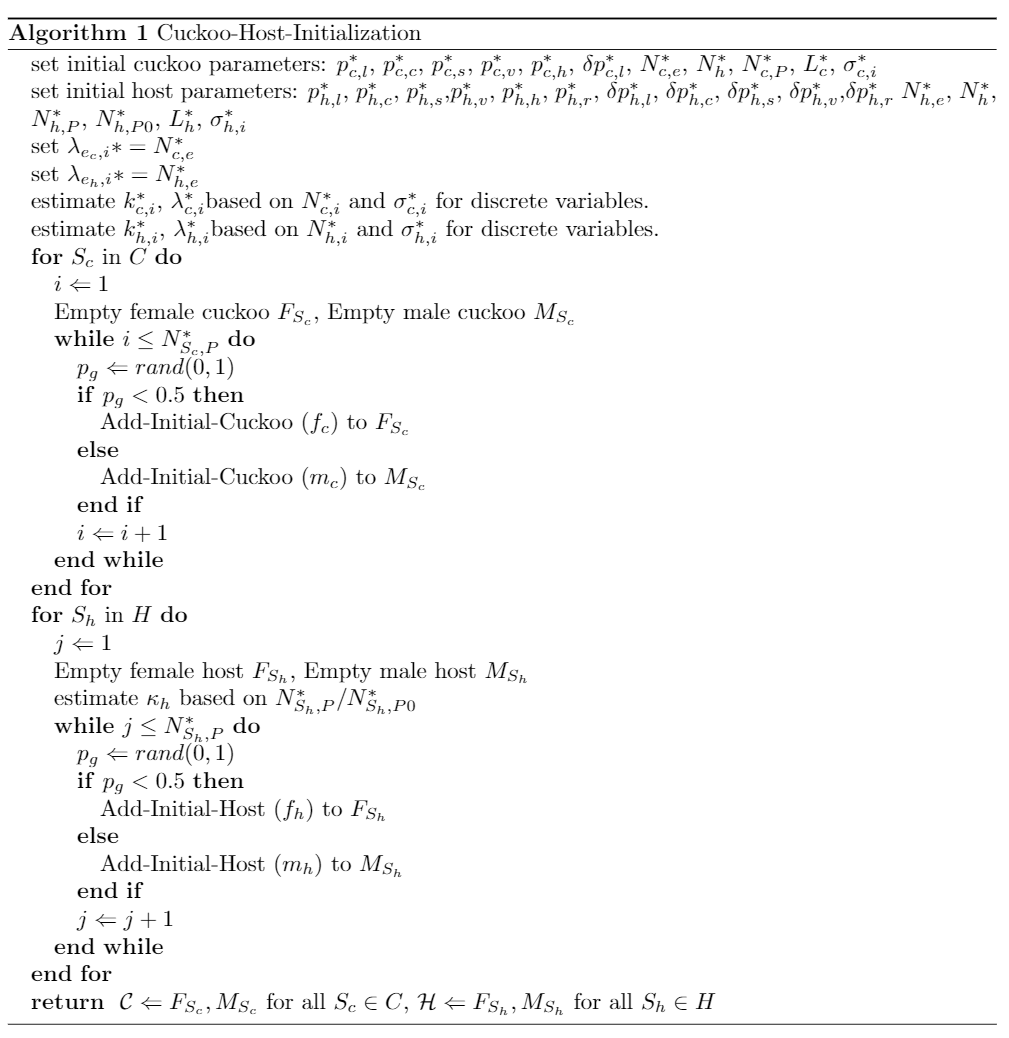


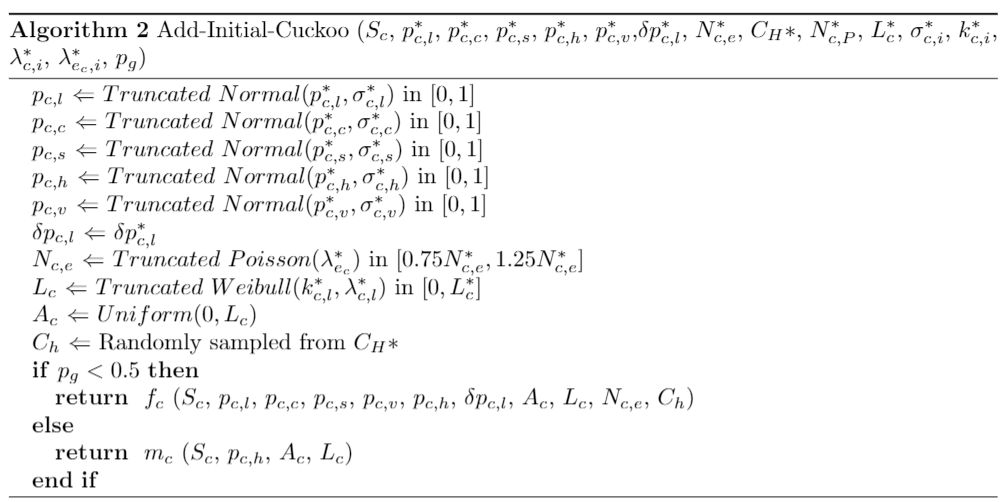


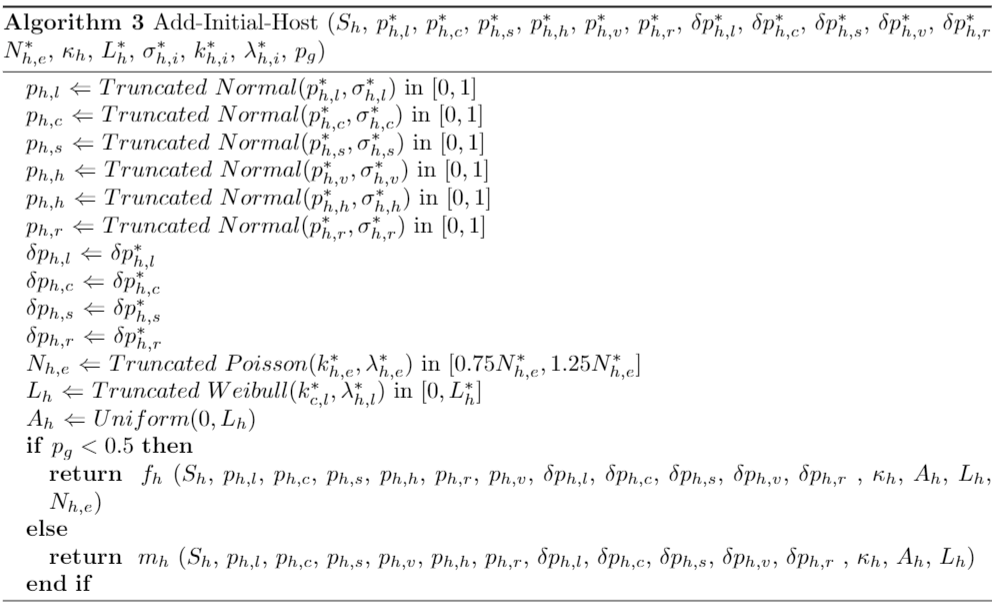


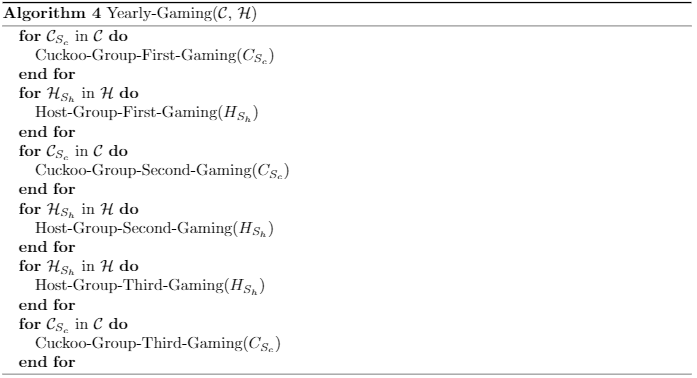


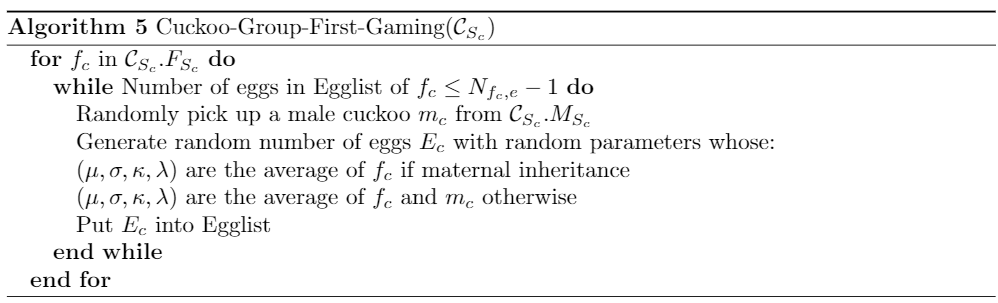


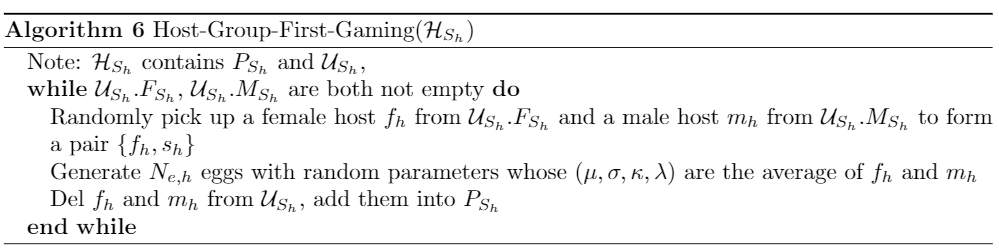


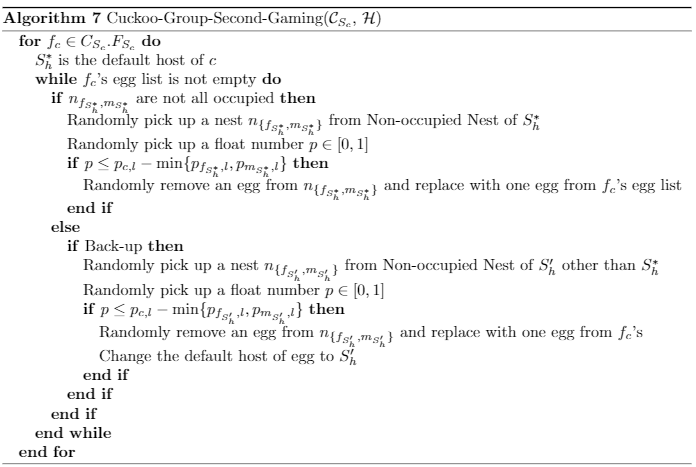


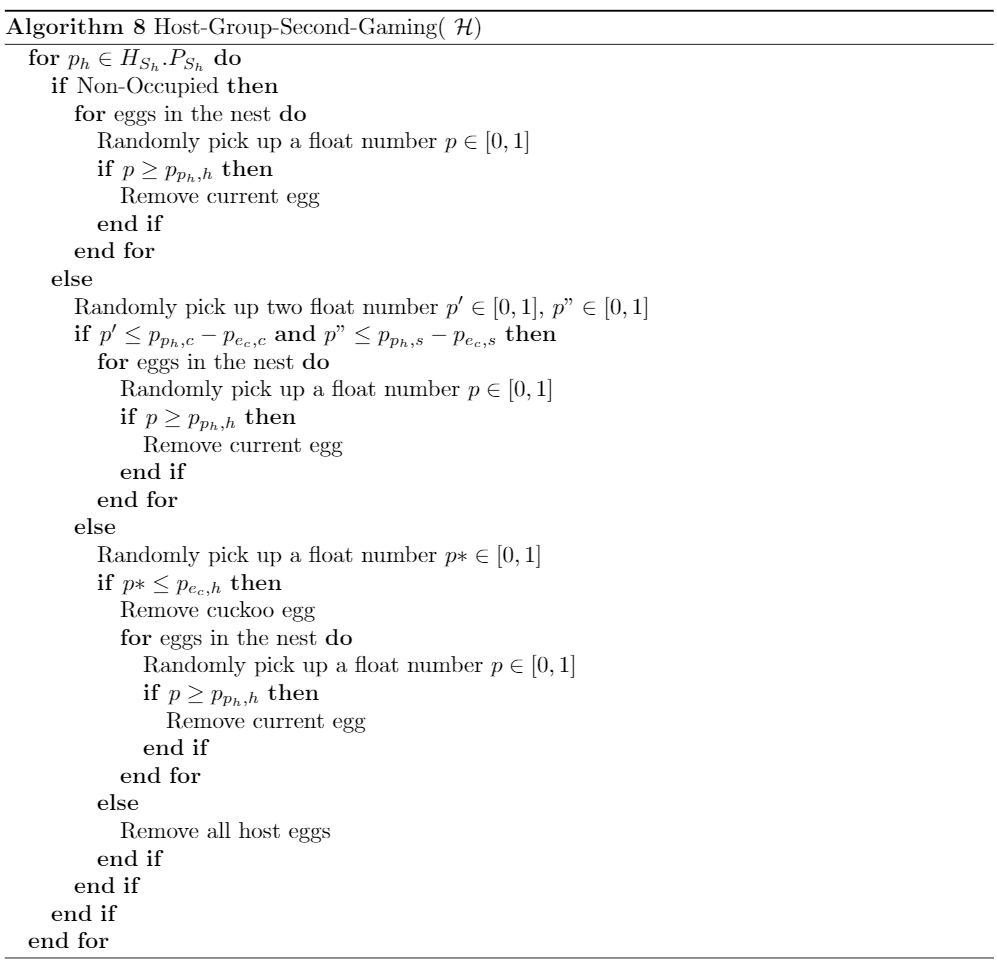


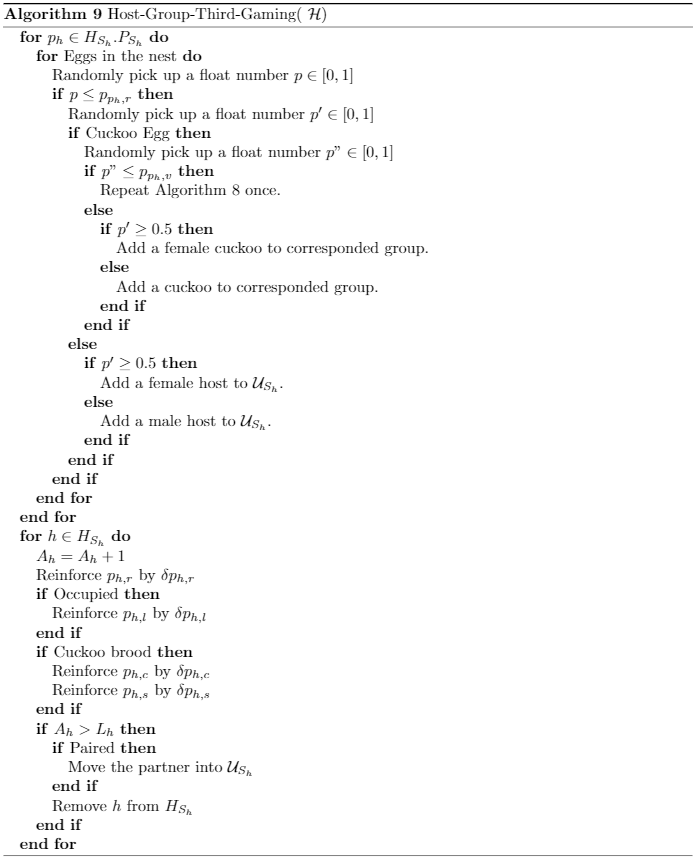


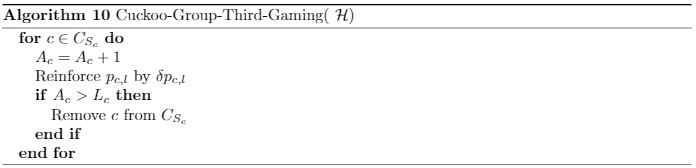

Supplement: Supplementary file 1 — Appendix S1. [file ECE3-15-e71721-s002.docx]
